# Single-cell atlas of peripheral blood mononuclear cells in pregnant women with hyperemesis gravidarum

**DOI:** 10.52601/bpr.2025.240064

**Published:** 2025-10-31

**Authors:** Ke Wu, Xueqiang Yao, Jie Wen, Haixi Hu

**Affiliations:** 1 Department of Radiology, the First Affiliated Hospital of USTC, Division of Life Sciences and Medicine, University of Science and Technology of China, Hefei 230001, China; 2 Department of Scientific Research, the First Affiliated Hospital of USTC, Division of Life Sciences and Medicine, University of Science and Technology of China, Hefei 230001, China

**Keywords:** Hyperemesis gravidarum, Pregnancy, scRNA-seq, Interferon, PBMCs

## Abstract

In early pregnancy, approximately 70% of women experience nausea and vomiting, with hyperemesis gravidarum (HG) can potentially lead to severe fluid and nutritional imbalances that require hospitalization. Although HG often resolves on its own in the early stages of pregnancy, its severity is linked to ketosis and elevated serum urea levels, as well as an increased risk of neurodevelopmental disorders in children. To further investigate the immune status of HG patients, we plan to conduct single-cell transcriptomic sequencing and plasma proteomic analysis of peripheral blood samples. This approach aims to elucidate the interactions and mechanisms of PBMCs and provide new insights into potential therapeutic interventions. Our findings indicate an increased proportion of neutrophils in HG patients, along with the upregulation of interferon genes and associated pathways. Notably, the activity of interferon-related TFs, such as STAT1, IRF7, and IRF9, was significantly elevated. Additionally, we observed a decrease in T cell activity in HG patients, while the functionality of NK cells and CD14^+^ monocytes was enhanced. The elevated plasma levels of NDEL1 may also have implications for fetal development. We have constructed a single-cell atlas of PBMCs from pregnant women with HG, which is expected to enhance our understanding of the immune response in HG and identify potential therapeutic targets for this condition.

## INTRODUCTION

Many women experience nausea and vomiting in early pregnancy, with an estimated incidence rate of up to 70% during gestation. Hyperemesis gravidarum (HG) is a severe form of nausea and vomiting that occurs during pregnancy. HG is associated with fluid, electrolyte, and acid-base imbalances, as well as nutritional deficiencies and weight loss, often necessitating hospitalization. Generally, approximately 60% of HG cases are resolved by the end of the first trimester, with up to 90% of cases resolving by 20 weeks of gestation (Gadsby *et al.* 1993). Research has concluded that ketosis and elevated serum urea are particularly effective laboratory indicators of the severity of HG (Fan and Yin 2024). In the United States, HG is a primary cause of hospitalization in early pregnancy and the second most common reason for hospitalization during gestation (Gazmararian *et al.* 2002). Until recently, there has been little significant advancement in understanding the molecular pathogenesis of nausea and vomiting in pregnancy (NVP) or HG (Fejzo *et al.* 2024). Current primary treatment methods largely rely on supportive measures until symptoms improve, which is part of the natural course of HG. This condition typically occurs with the progression of gestational age. However, studies have reported that severe and refractory cases can lead to significant adverse outcomes and may result in pregnancy termination (London *et al.* 2017). Research indicates that maternal HG levels are associated with an increased risk of neurodevelopmental disorders in children, including autism spectrum disorders and attention deficit hyperactivity disorder (Nijsten *et al.* 2022). These findings underscore the significance of investigating HG.

Recent studies have shown that both fetal production of GDF15 and maternal sensitivity to it significantly influence the risk of HG. Higher levels of GDF15 in maternal blood are associated with pregnancy and vomiting in HG (Fejzo *et al.* 2024). The neutrophil-to-lymphocyte ratio (NLR) can serve as a biomarker for HG (Beyazit *et al.* 2017). HG may be associated with an overactive immune response, potentially linked to the synthesis of pregnancy hormones (Minagawa *et al.* 1999). Studies have shown that cytokines derived from trophoblast cells can induce the secretion of hCG, leading to immune system dysregulation and enhanced humoral immunity (Heinrichs 2002). Consequently, HG may be caused by immune abnormalities during pregnancy.

Effective T cell activation requires the integration of three signals: the binding of antigen to the T cell receptor (first signal), interaction with co-stimulatory molecules (second signal), and activation by specific cytokine signals (third signal) (Chen *et al.* 2022). Additionally, research has shown that T cell activity decreases throughout pregnancy due to defects in the first and second signals (Chen *et al.* 2022). ISG15 (Interferon-Stimulated Gene 15) is an interferon (IFN)-inducible ubiquitin-like protein that plays a crucial role in the immune response. It is a member of the family of IFN-stimulated genes (ISGs) that are upregulated upon viral infections and other immune challenges. ISG15 is involved in modulating various immune processes, including inflammation, viral resistance, and cell signaling. It is known to conjugate to target proteins through a process called ISGylation, which is similar to ubiquitination, and thereby affects their stability and function. Given its key role in immune regulation and antiviral defense, ISG15 has been implicated in various diseases, including autoimmune disorders and cancer (Perng and Lenschow 2018). In this study, we focus on its potential involvement in HG to better understand its biological significance and therapeutic potential.

Currently, there are no studies specifically examining the alterations in the immune system of HG patients. We will conduct single-cell transcriptome sequencing and plasma proteomics on peripheral blood samples from HG patients to investigate their immune status. This approach will enhance our understanding of the interactions and mechanisms of peripheral blood mononuclear cells (PBMCs) in patients with HG. Ultimately, this study aims to gain a deeper understanding of the immune cell dynamics in HG patients, as well as the changes in protein content in the serum, and to explore potential therapeutic approaches.

## EXPERIMENTAL SECTION

### Inclusion and exclusion criteria

Inclusion criteria: Primiparous women aged 20–35 years, diagnosed with a singleton pregnancy, first pregnancy, no abortion, no drug abortion or ectopic pregnancy. Pregnant women registered in the First Affiliated Hospital of the University of Science and Technology of China at 6–12 weeks of gestation.

(1) Three HG pregnant women. Persistent vomiting not explained by other diseases, accompanied by one or more of the following symptoms: a) positive acute hunger indicators (typically urine ketones positive at 2+ or 3+；b) weight loss exceeding 5% of pre-pregnancy body weight; c) abnormalities in electrolytes, thyroid function, or liver function.

(2) Three normal pregnant women. No moderate to severe pregnancy vomiting episodes in the past seven days, assessed using the modified Pregnancy Nausea and Vomiting Assessment Scale (with scores of ≤6 indicating mild, 7–12 indicating moderate, and ≥13 indicating severe vomiting).

In addition, we included a pregnant woman with high urine ketone levels but no vomiting as a control to determine that the changes observed in HG are not influenced by elevated urine ketones.

Exclusion criteria. Individuals with known or suspected immune deficiencies, or those with uncontrolled infections; vomiting of non-pregnancy-specific origin due to other causes; women with molar pregnancies, multiple pregnancies, or those who have undergone assisted reproductive technology; individuals with other pregnancy complications such as gestational hypertension or gestational diabetes; those with a history of malignancies; patients taking specific medications; and individuals deemed unsuitable for inclusion in the study by the investigator.

### PBMC collection and treatment

Collect peripheral blood samples using two 2.5 mL anticoagulant tubes, gently rotating them to ensure thorough mixing. Transfer 1 mL of peripheral blood from one tube into a 1.5 mL centrifuge tube, and centrifuge at 2000*g* for 10 min to separate the plasma at 4°C, which should then be frozen at −80°C.

The remaining peripheral blood samples will be processed by Singleron. PBMCs were isolated using density gradient centrifugation with Ficoll-Paque Plus medium (GE Healthcare) and washed with calcium/magnesium-free PBS. To eliminate red blood cells, 2 mL of GEXSCOPE® red blood cell lysis buffer (RCLB, Singleron) was added and incubated at 25°C for 10 min. The mixture was then centrifuged at 500*g* for 5 min, and the pellet was suspended in PBS. Blood samples were centrifuged at 400*g* for 5 min at 4°C, and the supernatant was discarded. Following red blood cell removal, PBMCs were isolated by centrifuging at 400*g* for 10 min at 4°C. The supernatant was discarded, and the PBMCs were resuspended in phosphate-buffered saline to create a single-cell suspension.

Single-cell suspensions were converted to barcoded scRNA-seq libraries by using the Chromium Single Cell Library, Gel Bead & Multiplex Kit (10× Genomics), and following the manufacturer’s instructions. Briefly, cells were partitioned into Gel Beads in Emulsion in the ChromiumTM Controller instrument where cell lysis and barcoded reverse transcription of RNA occurred. Libraries were prepared using 10× Genomics Library Kits and sequenced on Illumina Nova6000 with 150 bp paired end reads by Singleron (China).

### Primary analysis of raw read data (scRNA-seq)

The raw base call (BCL) files were converted into FASTQ files using bsl2fastq. The quality of the droplet-based sequencing data was assessed using FastQC software. Subsequently, the reads were aligned to the GRCh38-2020-A human reference genome provided by Cell Ranger (version 7.0.0, 10× Genomics), and unique molecular identifier (UMI) counts were compiled for each gene in each cell.

### Data preprocessing

Scanpy (Wolf *et al.* 2018) v1.8.1 was utilized for quality control, dimensionality reduction, and clustering within Python 3.7. To reduce the influence derived from RNA contamination and doublets in the downstream analysis, DecontX (Yang *et al.* 2020) was used to estimate and remove contamination. The expression matrix was filtered to exclude cells with fewer than 200 gene counts or in the top 2% of gene or UMI counts, cells with over 5% mitochondrial content, and genes expressed in fewer than five cells. After filtering, 42,204 cells remained, with an average of 2103.4 genes and 5526.9 UMIs per cell. The data were normalized, log-transformed, and the top 2000 variable genes were selected. Batch effects between samples were eliminated using Harmony v1.0 (Butler *et al.* 2018). Principal Component Analysis (PCA) was performed using the top 20 components for clustering and dimensionality reduction. The Louvain algorithm (resolution = 1.2) was applied to group the cells into 22 clusters. Subsequently, UMAP was used for further dimensionality reduction and visualization of the clusters. For T cells and monocytes, Seurat 4.3.0 (Butler *et al.* 2018) was used for normalization, dimensionality reduction, and clustering, with t-SNE applied for further dimensionality reduction.

### Celltype annotation

The cell type identification of each cluster was determined according to the expression of canonical markers from the reference database SynEcoSys^TM^ (Singleron Biotechnology) (Zhang *et al.* 2023). SynEcoSys^TM^ contains collections of canonical cell type markers for single-cell seq data, from CellMakerDB, PanglaoDB and recently published literature.

### Differentially expressed genes (DEGs) analysis

To identify DEGs, we used the scanpy.tl.rank_genes_groups function based on the Wilcoxon rank sum test with default parameters. We selected genes that were expressed in more than 10% of the cells in either of the compared groups and had a Fold Change greater than 1.84 (log2 scale) as DEGs. Adjusted *p-*value was calculated by benjamini-hochberg correction and the value 0.05 was used as the criterion to evaluate the statistical significance.

### Pathway enrichment analysis

To investigate the potential functions of DEGs between group HG, HK and Control, Gene Ontology (GO) and Kyoto Encyclopedia of Genes and Genomes (KEGG) analysis were used with the “clusterProfiler” R package v 3.16.1 (Yu *et al.* 2012). Pathways with p_adj value less than 0.05 were considered significantly enriched.

### Subtyping of major cell types

To obtain a high-resolution map of NK, and Neutrophils, cells from the specific cluster were extracted and reclustered for more detailed analysis following the same procedures described above and by setting the clustering resolution as 0.5, 0.5.

For T cells and monocytes, cells from the specific cluster were extracted and reclustered for more detailed analysis following the same procedures of Seurat and by setting the clustering resolution as 0.6, 0.6.

### Analysis of single-cell pathway scoring

We used AddModuleScore for pathway enrichment analysis, a function from the Seurat package (Tirosh *et al.* 2016). This function calculates a score for each single cell by aggregating the expression levels of a given gene set and subtracting the aggregated expression of a randomly selected control feature set used as background. We also utilized AUCell to calculate the area under the curve (AUC) scores for all ranked genes (Aibar *et al.* 2017).

### Cell-cell interaction analysis

CellChat (version 0.0.2) (Jin *et al.* 2021) was used to analyze the intercellular communication networks from scRNA-seq data. A CellChat object was created using the R package process. Cell information was added into the meta slot of the object. The ligand-receptor interaction database was set, and the matching receptor inference calculation was performed.

### Single-cell regulatory network inference and clustering (SCENIC) analysis

We employed the R package SCENIC (version 1.3.1), along with RcisTarget (version 1.23.1) and AUCell (version 1.16.0), to examine the enrichment of transcriptome factors in cell subtypes (Aibar *et al.* 2017). Following the standard SCENIC procedure, we utilized the GENIE3 method (for a single sample) and GRNBoost (for the combined sample) to identify potential TF targets. Additionally, the activity of each regulon in each cell was assessed using AUCell, which integrates the expression ranks across all genes in a regulon and calculates the area under the recovery curve. We extracted neutrophils to analyze transcription factor differences between the three groups.

### Quantitative proteomics

Different samples underwent a series of processes including protein extraction, quantification, digestion, desalting, optional peptide fractionation, DDA for reference map establishment, DIA data collection, high-precision mass spectrometry detection, and database searching for qualitative analysis, followed by bioinformatics analysis. Using the iProteome one-stop data analysis cloud platform, we conducted database searches against the corresponding species database on the data files obtained from mass spectrometry, resulting in qualitative and quantitative protein outcomes. Additionally, we analyzed the distribution of protein quantification dynamic range and protein quantification density to assess the quality of the mass spectrometry data. Functional analysis and annotation of the identified proteins were performed, including GO enrichment analysis and KEGG pathway analysis.

## RESULTS

### Identification of cellular composition in peripheral blood mononuclear cells from patients with HG and healthy pregnant women

**Figure 1:**
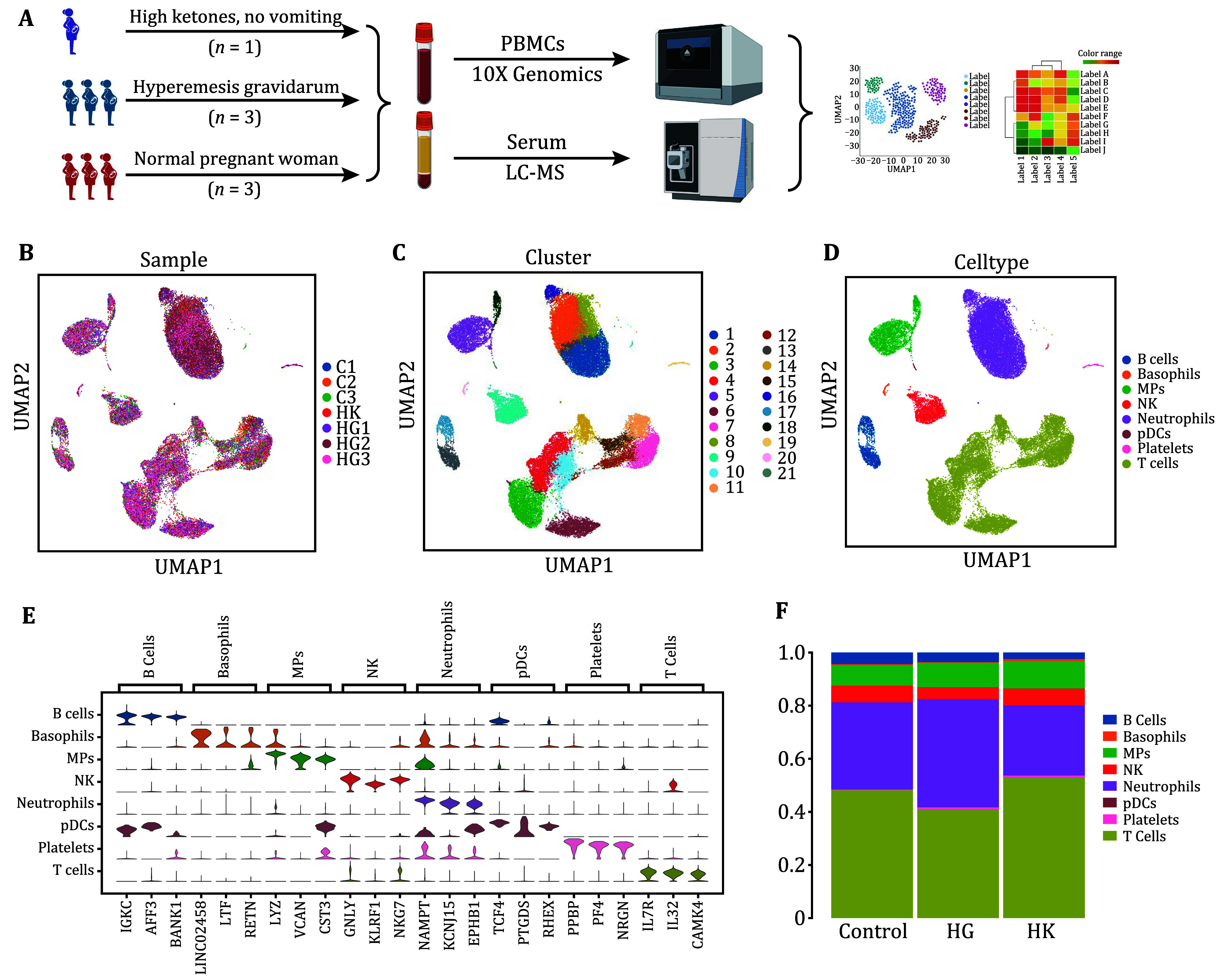
Single-cell gene expression profiling of PBMCs in healthy pregnant and HG pregnant women. **A** Schematic illustration of the study design. **B** UMAP representation of 41,682 high-quality single cells, where each cluster is assigned a distinct color to denote various sample identities. **C** UMAP illustration of the distribution across 22 clusters. **D** Through UMAP analysis of 41,682 high-quality single cells, we identified 8 main clusters of cell types, each differentiated by unique colors. **E** Violin plots displaying expression levels of marker genes specific to each cell type. **F** Proportions of each cell type in samples from women with HG and healthy pregnant women. Bars are colored according to cell type

To capture the transcriptional dynamics of PBMCs from patients with HG, we collected PBMC samples from three HG patients between gestational weeks 6 (GW6) and 12 (GW12), along with three healthy pregnancy samples as controls, and one pregnant woman with elevated ketones but not diagnosed with HG as a reference (HK). We also conducted proteomic sequencing on the plasma samples from these participants to analyze the disease from a multi-omics perspective (Fig. 1A). In transcriptome sequencing, after filtering the cells, a total of 41,682 cells were retained. These cells were annotated into eight major cell types using marker genes. These include B cells, basophils, monocyte macrophages, NK cells, neutrophils, pDCs, platelets, and T cells (Figs. 1B−1E). Most cell types are composed of cells derived from multiple samples, indicating that both HG patients and healthy pregnant women share a common composition of immune cells. Through the analysis of the ratios of these eight cell types, we found that the proportion of neutrophils increased and the proportion of T cells decreased in samples from pregnant women with HG (Fig. 1F).

### Proportion of ISG15-high neutrophil subpopulations, interferon pathway activation, and increased proliferation in HG patients

**Figure 2:**
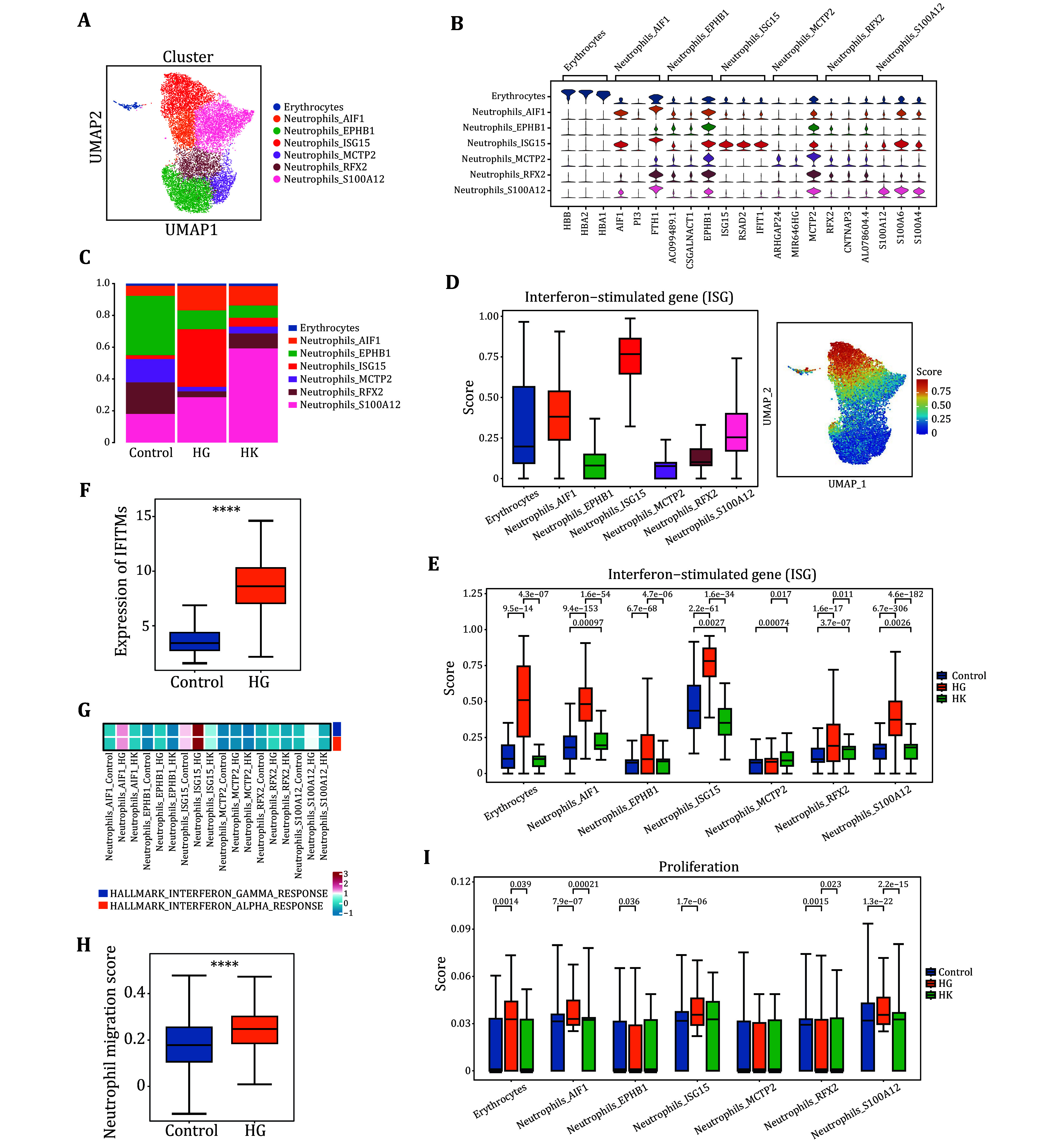
Characterization of neutrophils subpopulations in healthy pregnant women and women with HG. **A** We identified seven main clusters of neutrophils, each distinguished by unique colors. **B** Violin plots displaying expression levels of marker genes specific to each cell type. **C** Proportions of each neutrophil in samples from women with HG and healthy pregnant women. Bars are colored according to cell type. **D** Transcripts from neutrophils were assessed for enrichment of pathway in interferon-stimulated genes (ISG) comparing women with HG and healthy pregnant women. **E** Comparative assessment of the enrichment of each neutrophil transcript in the ISG pathways. **F** Comparative analysis of neutrophil transcripts among women with HG, healthy pregnant women, and pregnant women with elevated urinary ketones, assessing the enrichment of transcripts in the ISG pathways. **G** Heatmap displaying the assessment of different neutrophil types across each group using the Hallmark gene set. **H** Comparison of neutrophil transcripts between women with HG and healthy pregnant women, assessing the enrichment of transcripts in the neutrophil migration pathways. **I** Comparative analysis of each neutrophil type across the three groups, assessing the enrichment of neutrophil transcripts in the cell proliferation pathways. Student’s *t*-test was used to show the statistical difference between the two groups. Significance levels were defined as ns (not significant, *P* > 0.05), *P < 0.05, **P < 0.01, ***P < 0.001, and ****P < 0.0001

To further explore the transcriptomic changes in neutrophils from HG patients, we conducted a sub-clustering analysis of neutrophils and annotated them into seven subpopulations based on specific gene expression for each cluster (Figs. 2A and 2B). Analysis of the cell proportions in these seven subpopulations revealed a significant increase in the proportion of neutrophils expressing high levels of ISG15 in HG patients (Fig. 2C). We further analyzed the expression of interferon-related genes in different neutrophil subpopulations and found that neutrophils in the ISG15 high-expression subgroup exhibited significantly higher specificity in interferon-related gene expression (Fig. 2D). We hypothesize that the interferon expression levels in neutrophils of patients with HG are higher than those in healthy pregnant women. To validate our conclusion, we first examined the expression differences of interferon-related genes in neutrophil subpopulations across the three sample groups, finding that expression was elevated in each subpopulation (Fig. 2E). Next, we analyzed the expression of the interferon-related IFITM family in samples from women with HG compared to healthy pregnant women and found a significant increase in expression among the HG patients (Fig. 2F), further supporting our hypothesis. Additionally, we used the AddModuleScore function from the Seurat R package to calculate pathway scores based on the Hallmark gene sets, and obtained consistent results (Fig. 2G). Previous studies have shown that IFITM can inhibit trophoblast cell fusion, potentially leading to fetal harm (Buchrieser *et al.* 2019). Therefore, we speculate that the elevated IFITM levels in patients with HG may result in varying degrees of fetal damage. We then examined the differences in neutrophil function and found that the expression of genes related to migration and proliferation of neutrophils was elevated in patients with HG, indicating an enhanced activation of neutrophils (Figs. 2H and 2I).

### Ligand–receptor analysis reveals HG interactomes in neutrophils

**Figure 3:**
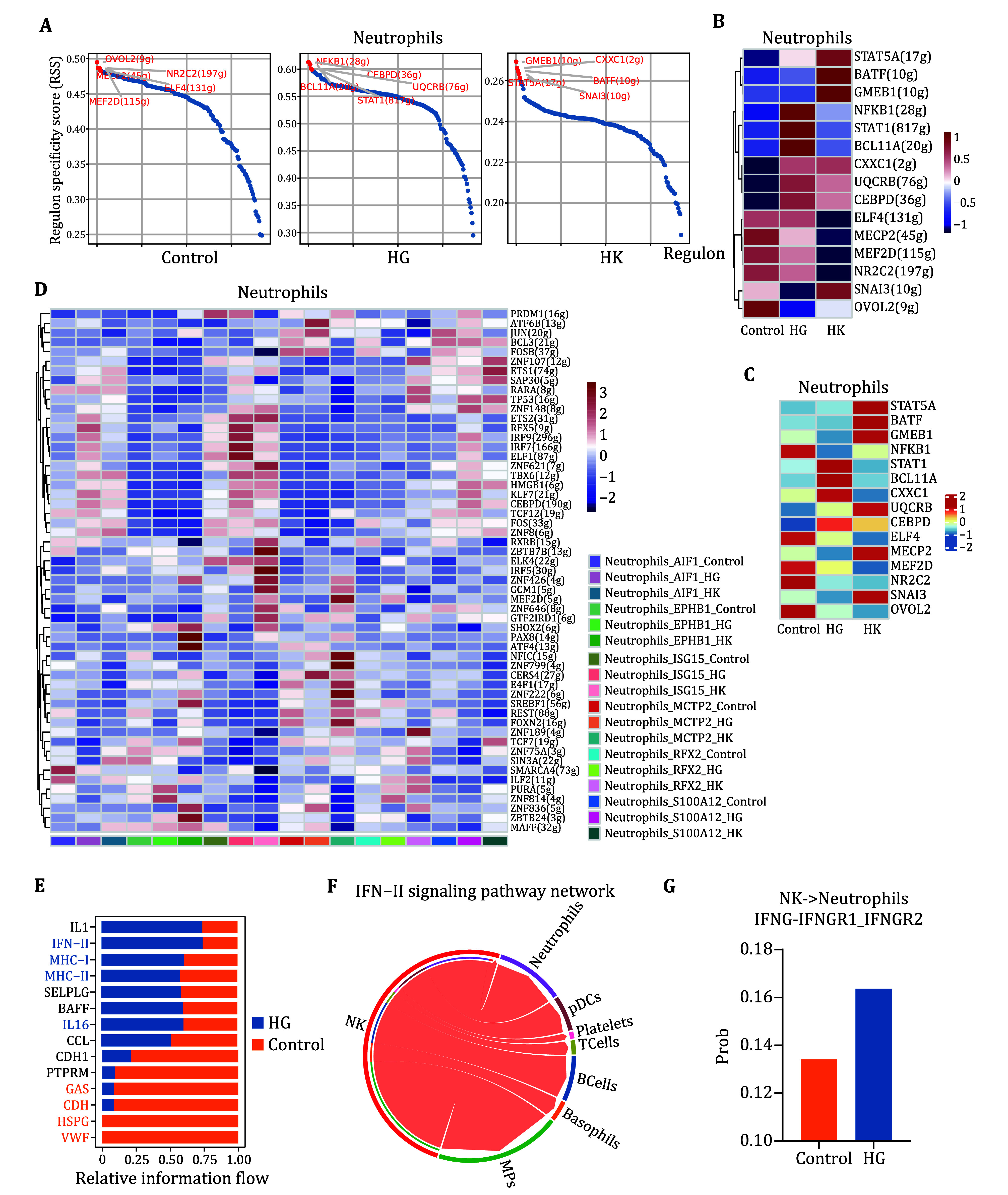
Transcription factors and cellular communication related to the interferon pathway in neutrophils of patients with HG. **A** Rank for regulons in neutrophils based on the RSS. **B** Heatmap of AUCell values for neutrophil TFs in the three sample groups. Red indicates upregulated TFs, while blue represents downregulated TFs. **C** Average expression levels of neutrophil TFs across the three sample groups. **D** Heatmap of AUCell values for neutrophil TFs across each neutrophil subtype in the three sample groups. Red indicates upregulated TFs, while blue represents downregulated TFs. **E** Bar chart ranking significant signaling pathways based on differences in overall information flow inferred between patients with HG and healthy pregnant women. Red bars represent pathways enriched in healthy pregnant women, while blue bars indicate pathways enriched in patients with HG. **F** CellChat analysis shows the quantity and strength of interactions among cell types involved in the IFN-II signaling pathway network. **G** Proportions of receptor-expressing cells mediating potential cellular communication between NK cells and neutrophils via the IFNG-IFNGR1_IFNGR2 pathway in healthy pregnant women and patients with HG

To further substantiate the differences in the interferon pathway in patients with HG, we conducted transcription factor analysis. By comprehensively considering the regulon activity score (RAS) and regulon specificity score (RSS), we identified STAT1 as the most potent Neutrophil_ISG15_HG-specific transcription factors (TFs), revealing elevated activity of the transcription factor STAT1 in the HG group, which is closely linked to the functional effects of interferon (Johansen *et al.* 2024) (Figs. 3A and 3B). Additionally, the expression level of STAT1 was found to be higher in these patients (Fig. 3C). Upon specifically comparing the activity of TFs among the neutrophil subtypes across the three sample groups, we observed that neutrophils from patients with HG, which showed high expression of ISG15, exhibited a distinct increase in the activity of the TFs IRF7 and IRF9 (Fig. 3D). Previous studies have shown that IRF7 and IRF9 are closely associated with the type I interferon pathway (Lu *et al.* 2018). Complex cellular responses are triggered by ligand-receptor binding and subsequent activation of specific signaling pathways. To determine the differences in molecular interactions among major immune cell types between patients with HG and healthy pregnant women, we employed CellChat for a bioinformatics analysis of intercellular communication. The overall signaling patterns identified by CellChat indicated significant upregulation of pathways associated with signaling and interferon production, particularly IFN-II (Fig. 3E), consistent with our previous findings. Furthermore, we identified specific ligand-receptor pairs involved in immune cell communication related to interferon, with a notable interaction between NK cells and neutrophils (Fig. 3F). Specifically, regarding the ligand-receptor pair IFNG-IFNGR1_IFNGR2, we found that the proportion of cells involved in this interaction between NK cells and neutrophils was significantly higher in patients with HG (Fig. 3G).

### Characterization of T cell subpopulations in patients with HG

**Figure 4:**
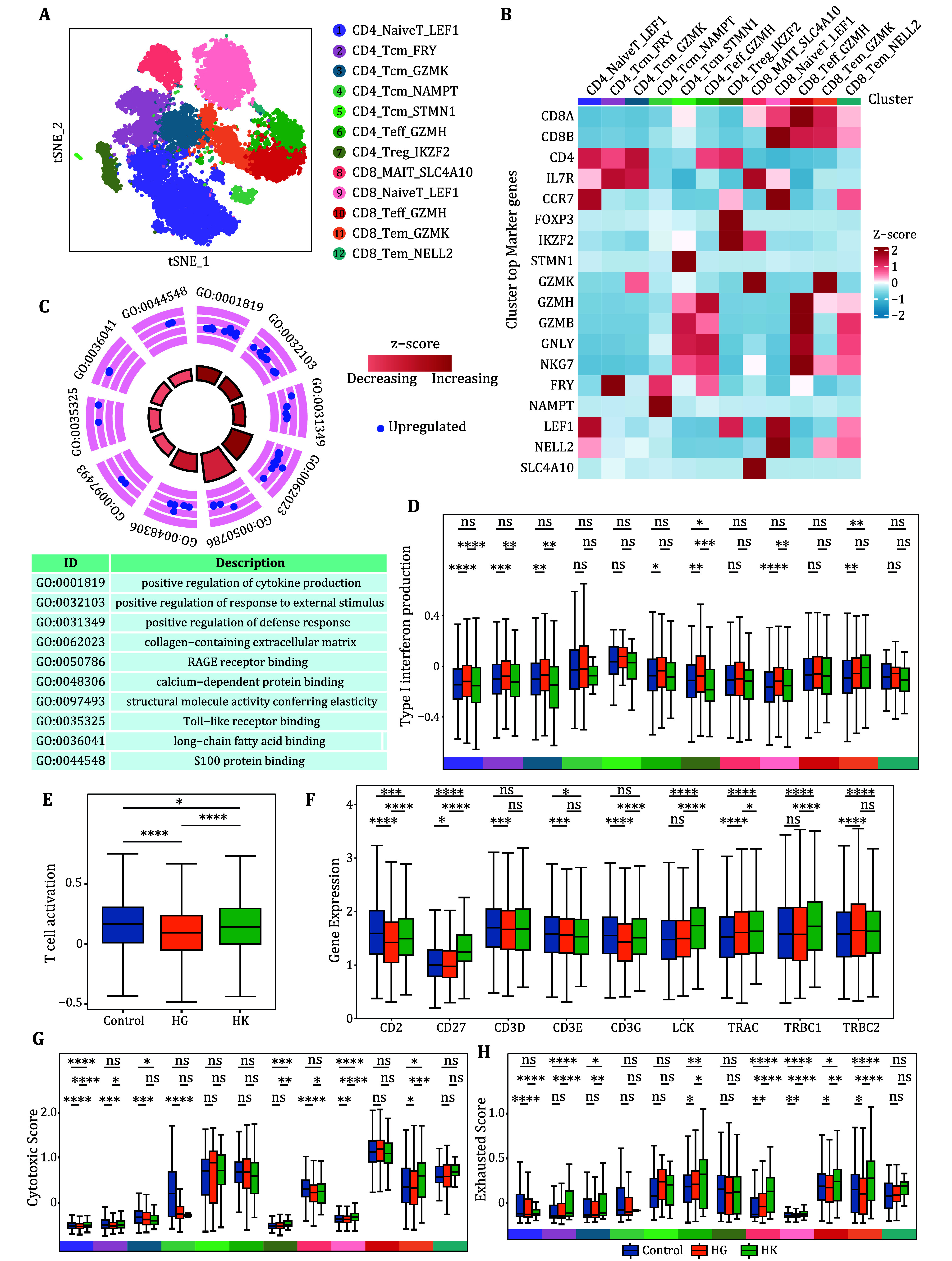


To further characterize the intrinsic structure and potential functional subtypes of the total T cell population, we performed dimensionality reduction of all T cells using UMAP. As a result, we identified 12 subclusters, including seven CD4^+^ cell subclusters and five CD8^+^ cell subclusters, each expressing its distinct set of characteristic genes (Figs. 4A and 4B). We aimed to investigate whether HG is associated with T cells and found that, compared to healthy pregnant women, the DEGs upregulated in T cells from patients with HG were primarily related to immune activation (Fig. 4C). Through the assessment of type I interferon production across T cell subpopulations in three groups of samples, we found that women with HG exhibit higher levels compared to healthy pregnant women (Fig. 4D), however, T cell activity remains relatively low (Crouse *et al.* 2015). Our findings demonstrate that T cell activity in patients with HG is lower than that of normal pregnant women (Fig. 4E). Consequently, we further examined the expression of genes related to the first and second signals, yielding similar results; these genes were significantly downregulated in HG patients (Fig. 4F). Therefore, we conclude that the first and second signals for T cell activation in women with HG are less effective compared to those in healthy pregnant women, resulting in reduced T cell activation overall. In addition, we assessed the cytotoxicity and exhaustion levels of each T cell subset across the three sample groups. Our findings indicate that T cell cytotoxicity is reduced in patients with HG, while exhaustion levels are also diminished (Figs. 4G and 4H).

### Immune landscape of B cells, NK cells and monocytes

**Figure 5:**
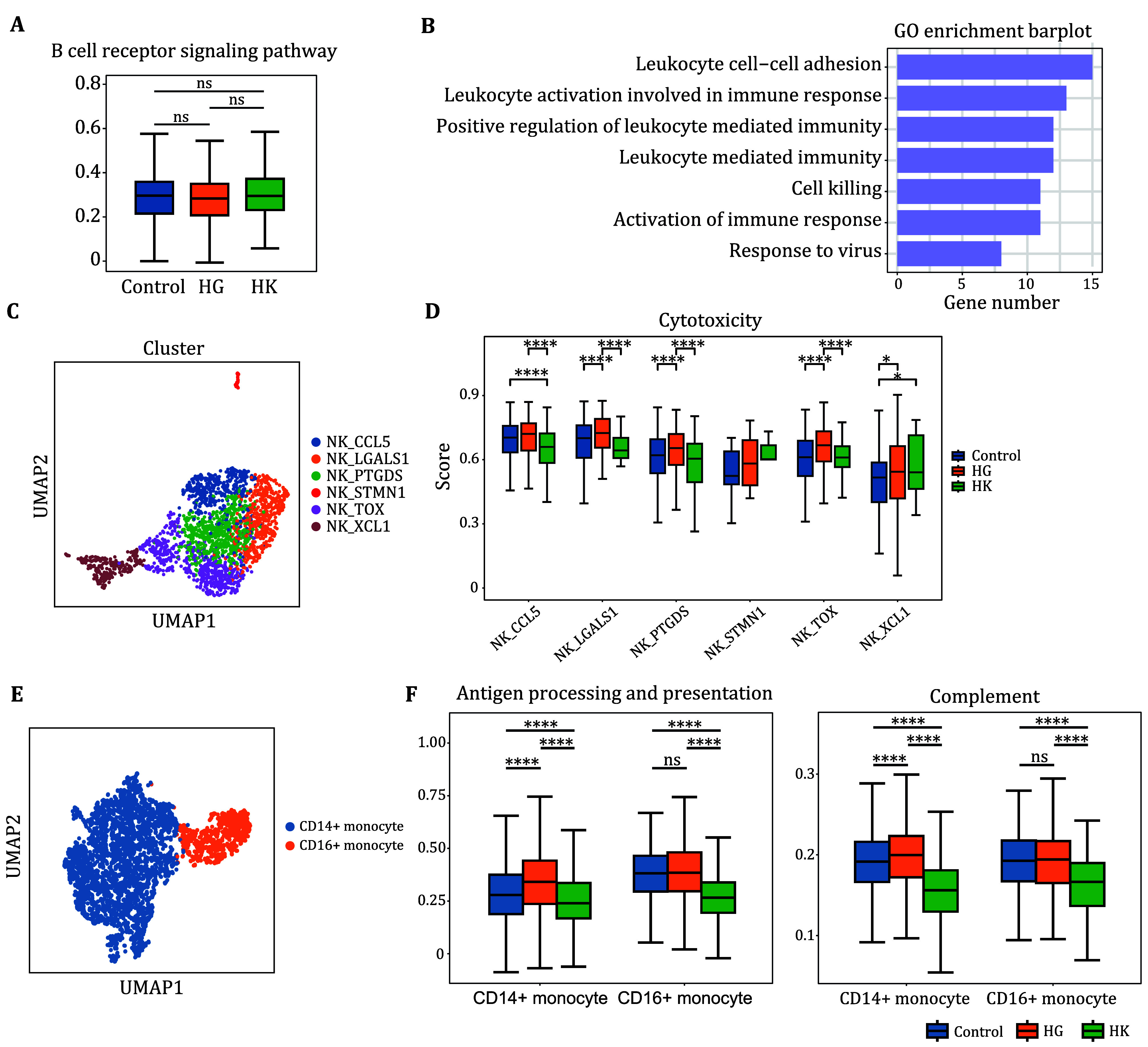
Subpopulation Characteristics of B Cells, NK Cells, and Monocytes. **A** Comparison of B cell transcripts between women with HG and healthy pregnant women, assessing the enrichment of transcripts in the B cell receptor signaling pathways. **B** GO analysis of DEGs in NK cells, highlighting pathway enrichment for upregulated genes. **C** We identified seven main clusters of NK cells, each distinguished by unique colors. **D** Comparative assessment of the enrichment of NK cell transcript in the cytotoxic genes. **E** We identified two main clusters of classical monocytes, each distinguished by unique colors. **F** Comparative assessment of the enrichment of monocyte transcript in the antigen processing and presentation and complement genes. Student’s *t*-test was used to show the statistical difference between the two groups. Significance levels were defined as ns (not significant, *P* > 0.05), *P < 0.05, **P < 0.01, ***P < 0.001, and ****P < 0.0001

Given our previous findings of differences in T cells and neutrophils between patients with HG and healthy pregnant women, we further investigated whether similar differences exist in other cell types. First, we observed no differences in B cell function (Fig. 5A). Next, we found that the upregulated genes in NK cells from patients with HG were enriched in pathways related to immune cell adhesion and cytotoxicity, indicating that NK cell activity is heightened in these patients (Fig. 5B). We further classified NK cells into six clusters and named them based on specific genes (Fig. 5C), revealing that cytotoxicity was significantly upregulated in each NK cell subgroup among HG patients (Fig. 5D). Lastly, we categorized classical monocytes into two groups: CD14^+^ monocytes and CD16^+^ monocytes (Fig. 5E). We found that the functional pathways associated with CD14^+^ monocytes were significantly upregulated in patients with HG (Fig. 5F), leading us to hypothesize that CD14^+^ monocytes may also be related to the disease.

### Proteomic analysis reveals NDEL1 as a potential contributor to developmental impairments in fetuses of HG patients

**Figure 6:**
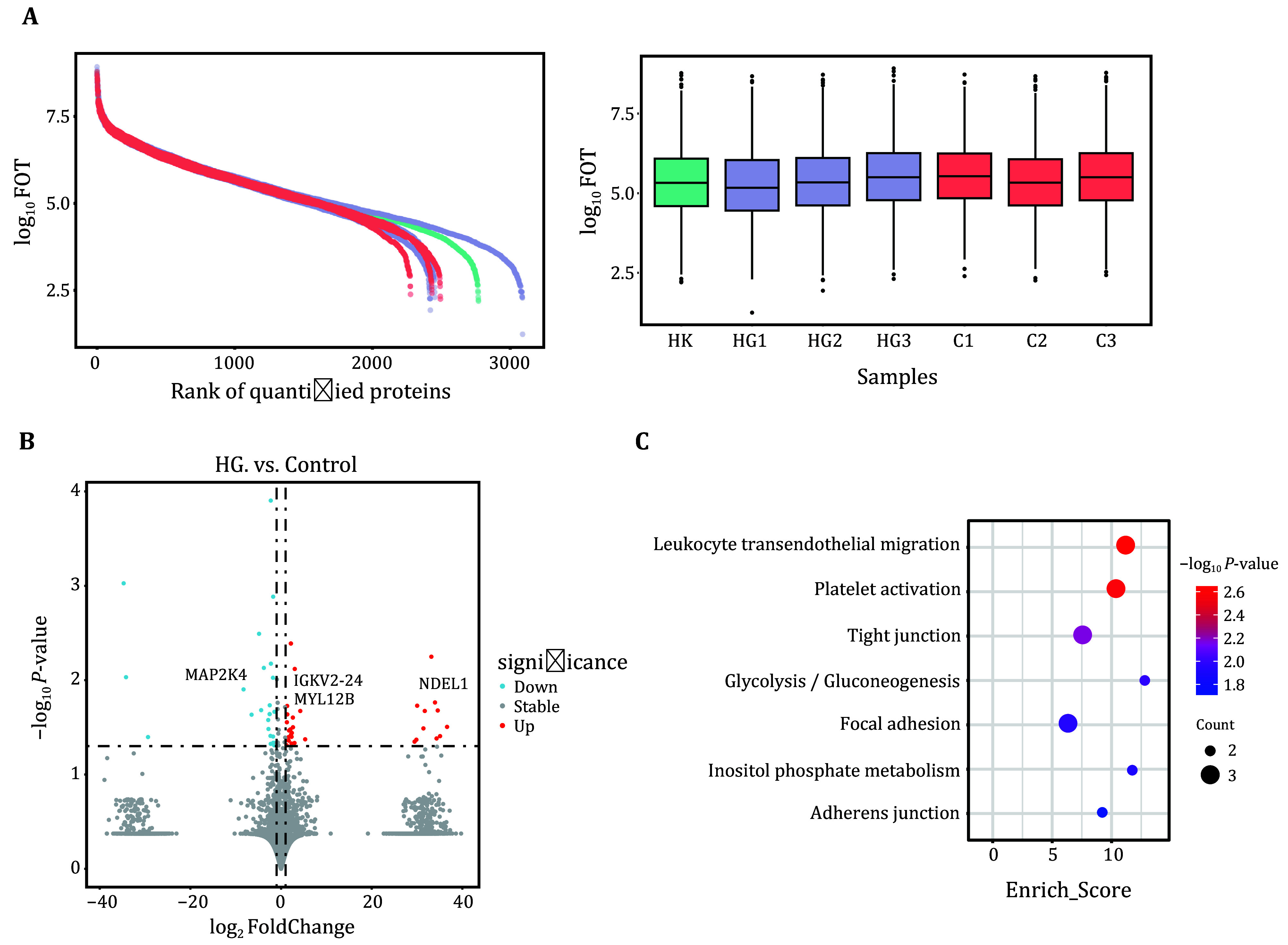
Proteomics analysis profile. **A** Distribution of protein quantification abundance dynamic range. The *x*-axis represents the ranked identified proteins, while the *y*-axis displays the quantification results of the identified proteins (log_10_) (left). Box plot of protein quantification results. The *x*-axis represents sample names, while the *y*-axis displays the protein quantification results (log10) (right). **B** Volcano plot of differentially expressed proteins (*P*-value < 0.05). The *x*-axis represents the fold change (log_2_), and the *y*-axis represents the significance of the differences (−log_10_ *P*-value). Red dots indicate significantly upregulated proteins, blue dots represent significantly downregulated proteins, and gray dots correspond to proteins with no significant differences. **C** Bubble plot of KEGG enrichment analysis for upregulated proteins. The size of the bubbles represents the number of proteins associated with each pathway, and the color indicates the significance level of the enrichment

We also processed the samples to isolate plasma for quantitative proteomics analysis. Initially, we examined the quantitative results of protein abundance ranges to assess the quality and reliability of the data, ensuring the accuracy of subsequent analyses (Fig. 6A). We then performed an analysis of the differential proteins between patients with HG and healthy pregnant women using the normalized data. This analysis identified 57 differential proteins, of which 24 were downregulated and 33 were upregulated. The upregulated proteins included NDEL1, IGKV2-24, MYL12B, among others (Fig. 6B). NDEL1 is a DISC1-interacting peptidase that can cleave neuropeptides, such as neurotensin and bradykinin, *in vitro*. It is also associated with neuronal migration and neurite outgrowth (Gadelha *et al.* 2016). Research has indicated that NDEL1 plays a crucial role in neurite outgrowth and neuronal migration during embryonic development. In our study, we observed an upregulation of NDEL1, which we hypothesize may lead to abnormalities in fetal brain development (Ye *et al.* 2017). We conducted an enrichment analysis of the upregulated proteins and identified pathways associated with enhanced immune activity, which further corroborates our previous findings (Fig. 6C).

## DISCUSSION

This study investigates the immune profile of PBMCs in patients during HG, offering new insights into the immune mechanisms involved in this condition. Our results show that HG patients have elevated neutrophil levels and upregulated interferon-related genes and pathways. Specifically, the activity of key interferon-related TFs, such as STAT1, IRF7, and IRF9, was notably higher. Additionally, T-cell activity was reduced in HG patients, whereas the functionality of NK cells and CD14^+^ monocytes was enhanced. Elevated plasma levels of NDEL1 may also have potential implications for fetal development. By generating a single-cell atlas of PBMCs from pregnant women with HG, this study contributes to a deeper understanding of the immune response in HG and helps identify potential therapeutic targets for future interventions.

Pregnancy is associated with significant systemic immune adaptations (Chen *et al.* 2022). However, we found that patients with HG exhibit a stronger immune response compared to healthy pregnant women. Previous studies on HG have primarily focused on clinical indicators, making it challenging to obtain a comprehensive understanding of cellular and molecular immune responses during pregnancy. To address this issue, we conducted a single-cell resolution analysis of the immune landscape of PBMCs in patients with HG.

During pregnancy, the maternal immune system undergoes changes to protect the allogeneic fetal tissue from premature rejection. In our study, we identified a significant impact of neutrophils in patients with HG. The upregulation of interferon-related genes and pathways in neutrophils supports our findings. While previous research has indicated that interferon levels increase during early pregnancy compared to healthy individuals, our study further reveals that these levels are even higher in cases of HG (Chen *et al.* 2022). Additionally, we found that in the neutrophils of patients with HG, the activity of interferon-related TFs STAT1, IRF7, and IRF9 is elevated compared to that of healthy pregnant women, further supporting our conclusions. This suggests an enhanced maternal immune response.

Additionally, in T cells, we observed that their activity is lower compared to healthy pregnant women. This reduction appears to be associated with a significant decrease in the expression of components of the TCR-CD3 complex (including CD3D, CD3E, CD3G, TRAC, TRBC1, TRBC2, and LCK) as well as costimulatory molecules (such as CD2 and CD27). These findings are consistent with previous research on T cell activity during pregnancy (Chen *et al.* 2022). Furthermore, in patients with HG, the cytotoxicity of T cells is reduced, and signs of exhaustion are also diminished. NK cells and monocytes also play a role in influencing the immune response associated with HG. The enhanced cytotoxic function of NK cells and the increased activity of monocytes indicate a heightened maternal immune response. However, in B cells, there are no significant changes in either proportion or function in HG patients, suggesting that HG is not related to B cell activity. Through the analysis of intercellular interactions, we found that NK cells communicate robustly with neutrophils within the type II interferon signaling pathway. We identified the ligand-receptor pair IFNG-IFNGR1_IFNGR2, which could potentially serve as a target for HG treatment. Through the analysis of plasma proteomics, we identified a significant upregulation of the protein NDEL1 in the plasma of individuals with HG. We hypothesize that the increased levels of NDEL1 may have an impact on fetal development.

This study has several limitations. The sample size of our data is relatively small, necessitating further research with larger samples to validate the identified changes in immune response associated with HG. Additionally, the observed changes in plasma and their potential effects require validation through animal studies. Future work should expand and confirm our findings by examining the immune changes in maternal PBMCs from the same women throughout their experience with HG and into the postpartum period.

To our knowledge, this is the first study to visualize the maternal PBMCs immune landscape in HG at single-cell resolution. This work is expected to enhance our understanding of the pathophysiological responses associated with HG.

## CONCLUSION

We observed an increased proportion of neutrophils in HG patients, along with the upregulation of interferon genes and related pathways. Notably, the activities of interferon-related TFs regulating neutrophils, such as STAT1, IRF7, and IRF9, were significantly elevated, suggesting these factors may serve as potential therapeutic targets for HG. Additionally, T cell activity was diminished in HG patients, while the functionality of NK cells and CD14^+^ monocytes was enhanced. The elevated levels of NDEL1 in plasma may also impact fetal development.

## Conflict of interest

Ke Wu, Xueqiang Yao, Jie Wen and Haixi Hu declare that they have no conflict of interest.
